# The Role of Anger Expression in Unmet Expectations and Depressive Symptoms

**DOI:** 10.1155/2023/8842805

**Published:** 2023-08-12

**Authors:** Izabela Kaźmierczak, Anna Zajenkowska, Joanna Rajchert, Adrianna Jakubowska, Agnieszka Abramiuk-Szyszko

**Affiliations:** Maria Grzegorzewska University, Poland

## Abstract

*Background and Objectives*. Depression is associated with unmet relational expectations, but little is known about how both partners experience meeting expectations and how this relates to anger expression and depressive symptoms. The aim of study 1 was to explore the role of anger expression in explaining the link between relational expectations and depression using the actor-partner interdependence mediation model. Additionally, social expectations beyond romantic relationships are associated with societal demands. Study 2 is aimed at investigating the role of anger expression in the relationship between internalized social demands (i.e., outer self-awareness) and depressive symptoms. *Design and Methods*. Online self-report data were collected from *N* = 194 romantic partners (97 dyads) in study 1 and *N* = 407 individuals in study 2. *Results*. In study 1, unmet expectations were associated with both actor and partner effects on depressive symptoms and anger expression. In particular, inwardly directed anger was linked to depressive symptoms in the case of the individual experiencing unmet expectations, whereas outwardly directed anger predicted such symptoms in the case of the partner's unmet expectations. In study 2, there was a positive association between outer self-awareness and directing anger inwards and outwards, which was linked to higher depressive symptoms. Furthermore, while directing anger inwards seemed to be a universal mechanism underlying the association, the interpersonal mechanism (i.e., directing anger outwards) was found to be dependent on gender.

## 1. Introduction

Unfulfilled expectations can arise in close relationships or within groups, such as family or society, to which an individual belongs. These interpersonal stressors can contribute to symptoms of depression through emotion dysregulation [1]. When there is a discrepancy between expectations and reality, it often leads to feelings of anger [2, 3]. This anger can be directed outwardly, towards others, or inwardly, towards oneself [4]. Difficulty expressing anger is linked to depression [5, 6], and angry outbursts are more common in people with depression, leading to the identification of a subtype of depression known as depression with anger attacks [7]. However, suppressing anger and feeling unable to express it also contributes to depression, and this effect can persist even after recovery [8]. Therefore, our project is aimed at examining both interpersonal and intrapersonal forms of anger expression as mechanisms underlying the relationship between unmet expectations and depressive symptoms.

We conducted two distinct research studies to test our hypotheses. The first study (study 1) involved dyads and focused on how people set and meet expectations in romantic relationships [9]. The second study (study 2) involved individuals and examined social expectations and the processing of self-information through the lens of society, or outer self-awareness [10]. By comparing the findings from these two studies, we can enhance our understanding of anger mechanisms in depression across different social contexts.

## 2. Study 1

Numerous studies [11–13] have shown that when there is a significant difference between an individual's expectations of their partner's behavior in a relationship and their actual behavior, it can lead to depression and also to angry feelings. However, to the best of our knowledge, prior research has failed to consider the potential association between the subjective experiences of both partners in meeting expectations and its possible link to depressive symptoms. Additionally, the role of anger expression in this association remains unclear.

### 2.1. An Actor-Partner Interdependence Mediation Model of Unmet Expectations and Depressive Symptoms through Anger Expression

According to the theory of interdependence [14], maintaining the relationship results in benefits for both partners, whether material or psychological (see also [15]). However, when one partner fails to fulfill the basic psychological needs of the other, it can result in feelings of frustration, irritation, and guilt. The partner whose expectations remain unmet may respond with disappointment and anger. If either partner struggles to effectively express their anger, ineffective anger processing can permeate the relationship, triggering a cycle of escalating anger, as noted by Liu et al. [16]. Elevated levels of anger or the suppression of anger can further contribute to the development of depression [6, 17] and an eventual increase in depressive symptoms over time [18]. Therefore, anger expression forms may serve as the pathways through which partners' unmet expectations lead to depressive symptoms. To explore this possibility, we utilized an actor-partner interdependence mediation model ([19], see also [20, 21]), which allows us to consider mediation, specific actor, and partner effects. Actor effects refer to situations where an individual's own data predict their outcomes, while partner effects occur when a partner's data can predict an individual's outcomes.

### 2.2. Unmet Expectations and Depressive Symptoms

#### 2.2.1. Actor Effects

When individuals experience a significant gap between their expectations and reality, or when promised rewards fail to materialize, they may display symptoms of depression [13, 22]. Furthermore, there is evidence to suggest that the frustration of psychological needs precedes the emergence of depressive symptoms over time [23]. Therefore, it is reasonable to hypothesize that *the less the individual's expectations regarding the partner are met, the higher the individual's depressive symptoms* (H1).

#### 2.2.2. Partner Effects

Developing stable and healthy self-esteem requires individuals to have positive and realistic expectations from their partners [24]. However, if these expectations become increasingly high and unattainable, individuals may experience negative evaluations of themselves and feelings of helplessness, which can contribute to the development of depression [25, 26], similar to low self-esteem [27]. Accordingly, *the less the partner's expectations are met, the higher the individual's depressive symptoms* (H2).

### 2.3. Unmet Expectations and Anger Expression

#### 2.3.1. Actor Effects

Unsatisfied needs and unmet expectations can naturally make individuals feel frustrated (e.g., [23]) and inclined to direct their anger at the failing partner. Conversely, the fear of being abandoned [28, 29] might suppress an open expression of disappointment [30]. In some cases, anger may be directed towards oneself rather than the partner who is failing to meet expectations, which can activate unconscious processes of turning anger inwards in response to loss ([31]; see also [32]). Hence, *the less the individual's expectations regarding the partner are met, the higher the individual's anger expression forms* (H3).

#### 2.3.2. Partner Effects

Even when individuals make genuine efforts to meet their partner's needs, their intentions may be doubted or the outcomes might fall short of satisfaction. As a consequence, feelings of guilt, frustration, and irritation can arise as individuals perceive themselves as lacking authentic support for their loved ones. In consequence, the individuals may either mask their annoyance, i.e., targeting anger inwards [33], or express it aggressively to the partner. Consequently, *the less the partner's expectations are met, the higher the individual's anger expression forms* (H4).

### 2.4. Anger Expression and Depressive Symptoms

#### 2.4.1. Actor Effects

Individuals with depression tend to experience anger more frequently [34] and at higher levels [6, 35] than those without depression. Psychoanalytic theorists propose that inwardly directed anger leads to feelings of guilt and contributes to the development of depression [36]. Research has further confirmed that depressed individuals tend to suppress their anger [8] and are more likely to engage in self-blame [37] or blame others [38]. These tendencies can persist even after recovery from depression [8], suggesting that targeting anger inwardly may contribute to vulnerability to depression. Overall, the evidence suggests that the expression of anger, particularly when directed inwardly, may play a primary role in the development and maintenance of depression. Hence, *the higher the individual's anger expression forms (particularly targeting anger inwards), the higher their depressive symptoms* (H5).

#### 2.4.2. Partner Effects

When a partner is disappointed and has difficulty managing their angry feelings, they may display irritability or even aggressive behavior. This can cause the other partner to question their ability to satisfy their partner and maintain a romantic relationship. Aggressive expressions of anger, whether verbal or physical, can lead to feelings of devaluation in the other partner, lowering their self-esteem and potentially leading to depression [39]. *That is why the higher the partner's ineffective anger expression (particularly directing anger outwards), the higher the individual's depressive symptoms* (H6).

### 2.5. Method

#### 2.5.1. Participants and Procedure

An a priori power estimation analysis was conducted with the APIMPoweR application [40]. Power estimation assuming that members in a couple are distinguishable, power = 0.80, *α* = 0.05; correlation between predictors, *r* = 0.50; and correlation between error terms = 0.15 indicated that 85 dyads would be needed to detect moderate direct actor and partner effects, *r*_partial_ = 0.30 (total *r* = 0.46). However, when dyad members would be indistinguishable, then 40 dyads would be enough to detect the effect of that size. Participants were 97 heterosexual couples recruited through a social media advertisement, who responded to an online questionnaire. Women were 17–58 years old, *M* = 25.14, SD = 7.29, and most of them completed secondary (58.8%) or higher education (39.2%). Men were 18–61 years old, *M* = 26.78, SD = 8.42, and also in most cases had secondary (55.7%) or higher education (37.1%). Relationships lasted between 1 month and 38 years, *M* = 61.68 months (5 years), SD = 86.88. The couples most frequently lived together but were not married (64%), less often were engaged (18.6%), or married (17.5%). The majority of couples lived in the city (81.4%). The study procedure was in accordance with ethical standards of the Declaration of Helsinki and approved by the university ethical committee.

#### 2.5.2. Measures

To measure depressive symptoms, we used the Hospital Anxiety and Depression Scale (HADS-M; [41]; Polish translation: [42]). The questionnaire includes 2 subscales referring to depressive symptoms and anxiety symptoms and consists of 14 items (7 for each subscale). Participants are asked to read each item (e.g., “I have sad thoughts”) and mark the appropriate answer that came closest to how they have felt during the last week. Items related to the depression subscale were summed up according to the key. The reliability of the depression aspect of the Polish version of HADS was acceptable (*α* = 0.78; [43]). In our research, Cronbach's *α* for the Depression Scale = 0.76.

To measure anger expression forms, we used the State-Trait Anger Expression Inventory (STAXI; [4]; Polish version) [44]. The questionnaire (57 questions based on multiple choice scale from ^“^hardly ever^”^ = 1 to ^“^almost always^”^ = 4) examines anger as a state, anger as a trait, and the expression of anger which is divided into anger-out (aggressively expressing anger towards others), anger-in (targeting anger inwards), anger control-out, and anger control-in (which apply to controlling angry feelings). For the purposes of this study, we focus on the anger-in scale (8 items, e.g., “I am angrier than I am willing to admit”; *α* = 0.87 [45] and the anger-out scale (8 items, e.g., “I argue with others; I say nasty things”; *α* = 0.86) [45].

The Expectation of the Partner Scale (SBOP; [46]) was used to measure the degree of expectations met by the partner. The questionnaire contains 18 items; the answers are rated on a scale from ^“^definitely not^”^ = 1 to ^“^definitely yes^”^ = 4. The questionnaire contains 3 scales: partners' similarity of attitudes towards the relationship (8 items, e.g., “He/she has a similar vision of our relationship as I do when it comes to legalizing it”; *α* = 0.92), matching worldview and values of partners (5 items, e.g., “His/her worldview on religion is similar to mine”; *α* = 0.75), and external characteristics and skills of the partner (5 items, e.g., “He can cook”; *α* = 0.84) [46]. All the scales contribute to the General Expectations Towards a Partner Scale (*α* = 0.73), which we focused on in our research.

#### 2.5.3. Analytical Approach

Data analyses were conducted using SPSS v. 26 for testing means and correlations and the actor-partner interdependence mediation model, AMIMeM [19], which uses structural equation modelling. We conducted the later analysis with an online application [47]. This model makes it possible to study the impact of a person's causal variable on their own outcome variable (actor effect) and on the outcome variable of the partner (partner effect). Those effects could also be mediated by a third variable pair (e.g., [48]). In the current study, three direct actor effects for each couple member were estimated: (1) expectation meeting was the predictor of actor anger-in (in one model) or anger-out (in the second model), and (2) actor anger was the predictor of actor depression. Finally, (3) actor expectations predicted actor depression. This allowed us to test for the actor-indirect effect of actor anger between actor expectations and actor depression (actor-actor indirect effect) and the effect of actor expectation on actor depression mediated through partner's anger (partner-partner indirect effect). However, also three partner direct effects for each couple member were estimated: (1) partner expectations on actor anger, (2) partner anger on actor depression, and (3) partner expectation on actor depression. Thus, the effect of partner expectation on actor depression mediated through actor anger (partner-actor indirect effect) but also actor expectation on partner depression mediated through actor anger (actor-partner indirect effect) could be estimated. All those effects are estimated for both members of the couple in case the dyad members would be distinguishable. In our study, dyad members are theoretically distinguishable (men and women in heterosexual relationships), but a test for empirical indistinguishability indicated that dyad members should be treated as indistinguishable as the model incorporating six equality constraints (assuming that all actor and partner effects do not vary due to gender) provided good fit (*χ*^2^(6) = 6.65, *p* = 0.354, RMSEA = 0.03).

### 2.6. Results

#### 2.6.1. Correlation Analysis

Results of zero-order correlations showed that the higher the meeting expectations and lower anger-out, the longer participants maintain the relationship. Meeting expectations also was negatively related to anger-in and anger-out and depression symptoms. Moreover, anger-in and anger-out were positively related to depression. Gender was not related to any of these variables.

We also conducted a zero-order correlation analysis between the man and woman scores within the same dyad. It showed that scores for anger-in and anger-out were not significantly associated (*r* = 0.14, *p* = 0.172, *r* = −0.01, *p* = 0.916, respectively). Depression symptoms were positively correlated, *r* = 0.186, *p* = 0.068, but this relationship was weak and only close to the accepted significance level of *p* = 0.05. However, meeting expectations was strongly and positively correlated (Cohen, 1988) in pair, *r* = 0.60, *p* < 0.001 (Table 1).

#### 2.6.2. Anger-In as Mediator

**Figure 1:**
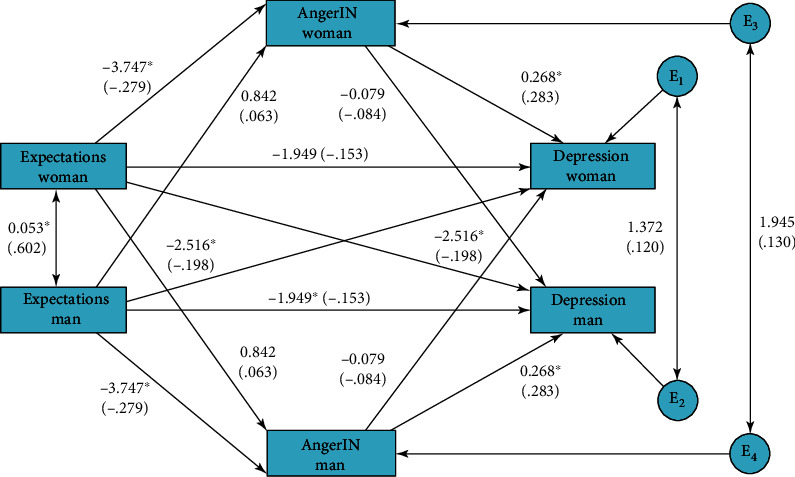
Unstandardized and standardized (in parenthesis) coefficients in the APIM testing the indirect effect of anger-in in the expectation-depression relationship. The roles were not empirically distinguished, so effects for men and women are the same.

Next, the APIM mediation was conducted in which anger-in was the mediator between meeting expectations and depression.  Figure 1 and Table 2 present the coefficients in this model.

Results indicated that actor meeting expectation had a negative effect on actor anger-in, and actor anger-in was positively related to actor depression. The direct effect of actor meeting expectations was also associated with actor depression, but this relationship was less strong and on the verge of being significant (*p* = 0.055). The total actor indirect effect was significant and accounted for 35% of the variance in the total effect of actor meeting expectations on depression. In this total indirect effect, the actor-actor indirect effect was more important (although not significant) than the partner-partner indirect effect, as it explained 33% of the total effect. Results regarding partner effects indicated that partner meeting expectations had direct negative effect on actor depression, but the total indirect partner effect was not significant, and neither were other partner effects.

#### 2.6.3. Anger-Out as Mediator

**Figure 2:**
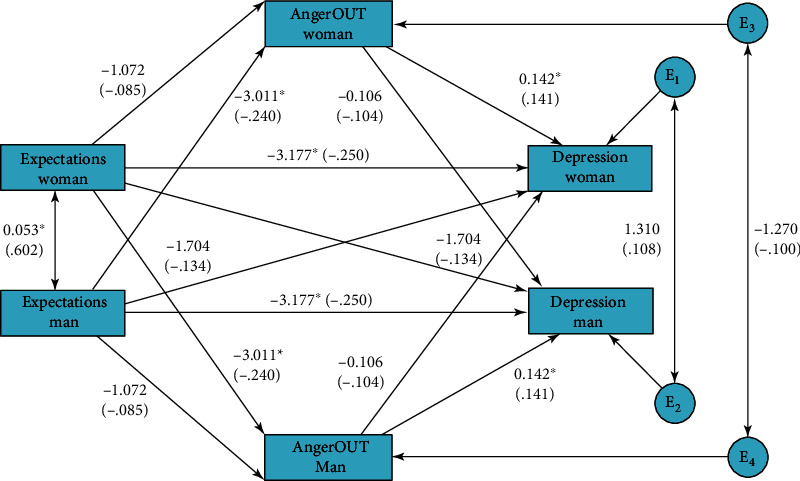
Unstandardized and standardized (in parenthesis) coefficients in the APIM testing the indirect effect of anger-out in expectation-depression relationship. The roles were not empirically distinguished so effects for men and women are the same.

The second model estimated the indirect effect of anger-out in meeting expectation-depression total effect. Coefficients for this model are presented in Figure 2 and Table 3.

Results of AMIMeM showed that the direct effect of actor meeting expectations on anger-out was not significant. However, higher actor depression symptoms were predicted by higher actor anger-out but also lower actor meeting expectation scores. Moreover, both the total and the direct actor effects of meeting expectations on depression were significant, whereas the total actor indirect effect through anger-out was not significant. The partner's direct effect of meeting expectations on the actor's anger-out was significant and negative, but no other partner's direct effects were significant. The partner total effect of meeting expectations on depression was close to the significance threshold (*p* = 0.054). The higher the partner meeting expectations was, the lower the actor depression symptoms. Nevertheless, the partner's indirect effect of meeting expectation on depression through anger-out was not significant.

### 2.7. Study 1: Result Summary and Discussion

Study 1 contributes to the existing literature on depression in the context of romantic relationships by examining the mediating role of anger in the association between unmet expectations and depressive symptoms. Our findings are consistent with prior research [13, 22] and demonstrate that unmet expectations, whether it be the individual's own expectations (H1) or their partner's (H2), are associated with increased depressive symptoms. Moreover, this study provides a unique contribution by revealing that the less the individual's expectations regarding the partner are met, the higher the individual's anger suppression, but not the expression of anger outwards (which only partially confirms our third hypothesis, H3). In turn, the less the partner's expectations are met, the higher the individual's expression of anger outwards but not inwards (H4). Unexpectedly, not only the higher the individual's anger suppression but also the higher the individual's directing anger outwards (H5), and the higher the partner's anger suppression (but not the partner's directing anger outwards) (H6), the higher the individual's depressive symptoms.

Finally, ineffective anger expressions served as a factor explaining the association between unmet expectations and depressive symptoms (H7). Specifically, inwardly directed anger was linked to depressive symptoms in the case of the individual experiencing unmet expectations, whereas outwardly directed anger predicted such symptoms in the case of the partner's unmet expectations. Hence, our findings suggest separate mechanisms of anger underlying relational expectations and depressive symptoms in both romantic partners. When individuals' essential needs in a relationship are not met, they may become less committed to the relationship [49], which is associated with depression and feeling angry at themselves for having needs that cannot be met [50]. Conversely, directing anger at the partner may involve fear of retaliation or rejection [6], as well as fear of damaging the relationship [8].

## 3. Study 2

In a broader sense, social expectations extend beyond romantic relationships and are intertwined with the demands of society, including social norms, rules, and moral standards [51]. Individuals who are highly socialized tend to derive their values from external sources, which can shape their social adaptation [52, 53]. Internalized social expectations can contribute to the development of outer self-awareness, wherein individuals engage in an objective evaluation of themselves by processing information through the societal lens. This outer self-awareness entails considering how one's thoughts, behaviors, and attributes align with the norms and standards imposed by society [10]. Therefore, we assume that the level of self-awareness development of an individual can be an indicator of their fulfilment of social expectations.

The research on the relationship between the outer form of self-awareness and depression is inconclusive. On one hand, internal information processing may increase both attention deficiency and negative mood in depression [54, 55]. In this case, the outer form of self-awareness may serve as a counteracting factor to the constant self-focused attention (e.g., [56, 57]) that is often associated with depression [10]. There is also evidence that positive mood enhances external information processing [58], and the latter decreases depressive mood [59, 60].

On the contrary, engaging in social comparisons often evokes negative emotions [61], unless individuals possess the necessary resources to meet those societal standards [62]. Difficulties in social adjustment can expose individuals to social feedback and pressure [63]. Regardless of the circumstances, violating social norms can trigger intense affective responses, including fear of authority or social exclusion, as well as feelings of guilt and self-blame, which can serve as motivators for behavioral change [64]. However, these emotions may also foster rumination on anger [65] and/or characterological self-blame [37]. Moreover, social comparisons have been consistently associated with depression across multiple studies (e.g., [66, 67]). Therefore, we stipulated that *outer self-awareness correlates with depressive symptoms* (H1); however, we did not assume whether the relationship was positive or negative. Moreover, *the relationship can be explained by the anger expression forms* (H2). Also, as there exist some gender differences in the use of self-blame and blame others [68, 69] and in the relationship between the use of these self-regulatory strategies and depression [70], we assumed that *the intermediary mechanisms of anger, underlying the outer self-awareness and depressive symptoms relationship, will occur or will be stronger in women* (H3).

### 3.1. Method

#### 3.1.1. Participants and Procedure

An a priori power estimation was conducted with the G^∗^Power application [71]. Assuming power = 0.80, *α* = 0.05, 366 participants would be needed to detect a small effect, *r*^2^_partial_ = 0.03 (*r* = 0.18), of each of the 3 predictors in the regression model. To detect a small, one-way correlation effect of *r* = 0.25, at least 95 individuals would be needed (e.g., men and women). Participants, *N* = 407, aged 18–66, *M* = 28.04, SD = 10.20, including 310 women (76.2%), and 97 men were recruited through social media advertisement and responded to an online questionnaire. A majority of them had completed higher education or were students, *N* = 273; 67%, fewer had graduated high school, *N* = 77, 18.9%; and minority had a lower level of education, *N* = 3. Participants most often lived in cities (70.5%). The procedure was in accordance with the ethical standards of the Declaration of Helsinki and approved by the university ethical committee.

#### 3.1.2. Measures

To measure depressive symptoms and anger expression forms, we used the same psychometric tool as in study 1. External self-awareness (ESA) was measured with the self-awareness scale [10]. The questionnaire consists of 80 items and contains four scales corresponding to four forms of self-awareness: individual, defensive, outer, and reflective. The external self-awareness scale contains 15 items (e.g., “I like to know what people around me are talking about” or “When making a decision I think of what my friends are going to say about it”) and has satisfactory reliability, *α* = 0.78. Apart from the ESA scale, the tool distinguished the defensive self-awareness scale (25 items, e.g., “I have an uneasy feeling that I am not as worthy as other people”; *α* = 0.91), the personal self-awareness scale (23 items, e.g., “My behaviour is influenced mainly by my judgments and attitudes”; *α* = 0.80), and the reflective self-awareness scale (17 items, e.g., “Life is an unending examination”; *α* = 0.88).

#### 3.1.3. Analytical Approach

We tested the moderated mediation model with the PROCESS macro [72], which uses OLS (ordinary least squares) regression. We included external self-awareness as the predictor variable for depression symptoms. Anger-in and anger-out were mediators in this model, and gender moderated the ESA effect on both anger scores. We also tested moderated mediation effects by comparing the indirect effects for men and women [73].

### 3.2. Results

#### 3.2.1. Correlational Analysis

The zero-order correlation analysis indicated that women were higher on ESA and anger-out than men, but there were no significant gender differences in anger-in and depression. Furthermore, ESA was positively related with anger-in, anger-out, and depression. Also, anger-in and anger-out were positively associated with depressive symptoms. Correlation indices and means for study variables are presented in Table 4.

#### 3.2.2. Mediation Analysis

Next, 2 moderated mediation models were tested for anger-in and anger-out as mediators and gender as a moderator of the ESA effect on anger. Both models predicted a significant amount of variance in depression; the model for anger-in predicted 7% of the variance, whereas the model for anger-out predicted 6% of the variance. Gender had the main negative effect on anger-out (women were higher on anger-out than what was already indicated in the correlation analysis). Gender was also a significant moderator of the ESA effect on anger-out but did not shape the ESA effect on anger-in. Coefficients for both models are presented in Table 5.

Simple slope analysis of the ESA effect on anger-out showed that ESA was positively related to anger-out in men, *B* = 0.16, SE = 0.05, *t* = 3.52, *p* < 0.001, 95% CI (0.07; 0.25), but not in women, *B* = 0.01, SE = 0.03, *t* = 0.53, *p* = 0.597, 95% CI (-0.04; 0.07).

In the next step mediation effects of anger-in and anger-out were analysed. Depressive symptoms were related to both anger expression forms. Regression coefficients for the model predicting depression are presented in Table 6.

The total effect of ESA on depression was positive and significant, *B* = 0.10, SE = 0.02, *t* = 4.30, *p* < 0.001, 95% CI (0.05; 0.14), *r* = 0.210. When anger-in and anger-out were included, the direct effect was no longer significant, and 56.7% (*r* = 0.12) of the total effect was mediated through anger. Both anger expressions were significant mediators. The indirect effect through anger-in was *B* = 0.04, boot SE = 0.009, 95% CI (0.03; 0.06), *r*_partial_ = 0.09, and through anger-out was *B* = 0.01, boot SE = 0.005, 95% CI (0.002; 0.02), *r*_partial_ = 0.02. Moreover, the conditional indirect effect of anger-out was significant only in men, *B* = 0.03, boot SE = 0.01, 95% CI (0.008; 0.05), but was not significant in women, *B* = 0.002, boot SE = 0.005, 95% CI (-0.007, 0.01), and the difference between those effects was significant, index = 0.02, boot SE = 0.01, 95% CI (0.006; 0.05). However, the indirect effect through anger-in was not moderated by gender, index = 0.02, boot SE = 0.02, 95% CI (-0.02; 0.06), and both indirect effects, for women, *B* = 0.04, boot SE = 0.01, 95% CI (0.02; 0.06), and for men, *B* = 0.06, boot SE = 0.02, 95% CI (0.03; 0.09) were significant.

### 3.3. Result Summary and Discussion

Study 2 complements the previous one by shedding a new light on the specific role of internalized social expectations (i.e., outer self-awareness) by determining their positive relationship with depressive symptoms (H1). This aligns with the research indicating that failing to meet social expectations can contribute to the development of depression [74, 75].

Further, it revealed that both forms of anger expression accounted for the relationship (H2). As expected, we found that the higher the level of outer self-awareness, the higher the tendency to direct anger both inwards and outwards which also coexist with higher depressive symptoms. This suggests that some highly socialized people who are unable or unwilling to meet particular societal expectations may experience some difficulties regulating their emotions. They may experience frustration and anger, particularly when concerned about negative judgments from others [76, 77]. As a result, these individuals may direct their anger inwardly due to the fear of expressing it and potential rejection [6] leading to depression. To further validate this assumption, future studies should consider including measures of fear of rejection or sensitivity to rejection. Conversely, these individuals can direct their anger outwardly, which has also been linked to depression [78]. Therefore, it would be interesting to observe and investigate individuals engaged in protest activities characterized by heightened emotional expression as a means of externally addressing social issues [51, 79]. These observations would provide valuable insights into the role of externalized anger and its relationship with depressive symptoms in the context of social activism.

Interestingly, the mechanism of anger expression forms underlying the relationship between outer self-awareness and depressive symptoms was dependent on gender (H3). While both targeting anger inwards and outer self-awareness were related to depressive symptoms among men and women alike, targeting anger outwards was not related to depressive symptoms among women (although they scored higher than men on both outer self-awareness and targeting anger at others), which is contradictory to our hypothesis.

## 4. General Discussion

The current project focused on the relationship between meeting expectations, depression, and anger expression forms in two research contexts: interpersonal (study 1) and social (study 2). Consistently across the studies, both anger expression forms were associated with depressive symptoms (studies 1 and 2), and they mediated the relationship between meeting relational (study 1) or social (study 2) expectations and depressive symptoms.

Additionally, in study 1, we identified separate anger mechanisms underlying relational expectations and depressive symptoms for both romantic partners. Most importantly, when an individual's expectations are not met and when they suppress their anger, they also develop depressive symptoms. However, when they do not meet their partners' expectations, then directing anger outwards coexists with their depressive symptoms. A person whose important needs are not being met over time may assume an attitude of being less committed to the relationship [49]. Both lower commitment [50] and unmet needs [80] are associated with depression and may also relate to anger at oneself for the mere fact of having these needs, especially in the face of unsuccessful attempts to satisfy them. Interestingly, the individual's anger directed outwards was not related to the partner's depression. Therefore, it could be hypothesized that this form of anger expression serves as a neutral (if not protective) factor in predicting depression within the dyad in this particular context. By expressing anger towards the external source, individuals may potentially alleviate negative emotions associated with not being able to meet some partners' expectations, thus reducing the likelihood of developing their own depressive symptoms. This outward expression of anger may serve as a mechanism for releasing pent-up frustration and maintaining a healthier emotional balance within the relationship. Additionally, there is a need for longitudinal studies that examine the dynamic interplay between changes in relational expectations, the expression of anger (anger loop), and the development of depressive symptoms in time. By investigating these variables over an extended period, researchers can gain a deeper understanding of how fluctuations in expectations and anger expression forms within the relationship relate to the onset and progression of depression symptoms. Longitudinal studies would provide valuable insights into the temporal nature of these associations and help establish a clearer causal relationship between these factors.

The actor-partner perspective applied in study 1 does not allow to distinguish potentially specific effects for men and women. Gender difference perspective was provided in study 2, where mediation through targeting anger outwards occurred in men only (targeting anger outwards was not significantly associated with depression in women). This may reflect differences in gender socialization, as women may be more socialized to express their emotions in ways that are less likely to lead to outward displays of anger [81]. What is more, although separate gender role expectations allow men to relatively openly express their anger [82, 83] in the face of boundary crossing, a man may react in a more or less adaptive way. Aggressive forms of anger expression (such as arguing with others or saying nasty things) when a man considers some social demands too difficult or impossible to meet do not effectively protect self-esteem and are a risk factor for interpersonal conflicts that can lead to depression [84].

On the other hand, some women may suppress expressions of anger due to their fear of being considered socially unacceptable [83]. Directing anger inwards may be the consequence of their tendency to meet others' expectations, which is consistent with the finding on higher outer self-awareness in this group. The primary emotion of anger aroused by excessive social demands may be accompanied by the secondary emotion of anger that arises due to the perception of limited possibilities for expressing feelings. In consequence, strong emotional loading in combination with difficulties in emotion regulation may lead to self-blame or unverbalized blaming of others (see [38]) and thus to depression.

## 5. Limitations

There are some limitations to the current studies that need to be acknowledged. First and foremost, while certain findings suggest that anger expression tendencies may be causally related to depression, it is highly recommended to draw conclusions regarding causality based on correlational cross-sectional studies with caution. Furthermore, replicating the model among individuals diagnosed with depression would enhance our understanding of the anger management mechanism in the context of meeting relational and/or societal expectations. It should be also noted that in the case of study 1, the power estimation did not include indirect effects. Moreover, the correlation between predictors was lower than assumed. Thus, replication of study 2 with more numerous sample would allow for detection of less strong indirect effects.

## 6. Conclusions and Practical Implications

Our studies delved into two distinct research contexts, namely, interpersonal and social, to enhance our understanding of the connection between meeting expectations and depression. In the context of romantic relationships, we observed a significant role played by inwardly directed anger in depression when individuals' relationship expectations are not met. However, when they do not meet their partners' expectations, then directing anger outwards coexists with their depressive symptoms. In turn, in the realm of societal expectations, the higher the levels of outer self-awareness, the stronger the anger directed inwardly and outwardly, and both anger expression forms lead to depressive symptoms.

To further illuminate these findings, future research may investigate the childhood experiences and their relationship not only with anger expression forms and meeting expectations but also with such factors as fear of rejection or sensitivity to rejection. It is worth noting that individuals with depression often originate from families lacking emotional support and care [85]. Growing up in such an environment may contribute to the development of introjective/self-critical depression, characterized by feelings of guilt, worthlessness, and anger directed towards oneself [86]. These emotions are often accompanied by a fear of rejection from caregivers, which can later translate into a fear of rejection by romantic partners in adulthood [85].

Finally, the findings suggest the importance of psychoeducating patients with depression about safe experiencing and expressing of anger, without directing it towards themselves or exhibiting anger outbursts towards others. These results can also be applied in couple therapy by incorporating the anger reinforcement mechanism (anger loop) and its maladaptive forms of expression into the conceptualization of the couple, as well as linking them to the severity of depressive symptoms in both partners, albeit in a distinct manner depending on whose needs are unfulfilled. Furthermore, the results highlight the need to provide support to patients in romantic relationships to express anger in situations where their partner's expectations cannot be met, as well as deepening the understanding of cognitive mechanisms through which patients direct anger inwards when their own needs cannot be satisfied by their partner.

## Figures and Tables

**Table 1:** Means and zero-order correlations between study variables.

	1.	2.	3.	4.	5.	6.
1. Gender	—	0.000	-0.041	-0.028	-0.116	0.018
2. Duration		—	0.151^∗^	-0.013	-0.169^∗^	0.074
3. Expectations			—	-0.240^∗^	-0.223^∗^	-0.332^∗^
4. Anger-in				—	0.061	0.330^∗^
5. Anger-out					—	0.232^∗^
6. Depression						—
*M*	—	61.66	3.35	18.59	16.39	11.64
SD	—	86.62	0.29	4.01	3.77	3.79

^∗^ *p* < 0.05; gender coded: 1, woman; 2, man. Duration, relationship duration.

**Table 2:** Effects of the mediation model testing the direct and indirect effects of anger-in in meeting expectation-depression relationship for partner (P) and actor (A).

Effect	Type	Estim.	*p*	95% CI	Stand.
Direct expectation → anger-in	A	-3.75	<0.001	-5.92; -1.56	-0.28
	P	0.84	0.451	-1.34; 3.03	0.06

Direct anger-in → depression	A	0.27	<0.001	0.14; 0.39	0.28
	P	-0.08	0.217	-0.20; 0.04	-0.08

Direct expectations → depression	A	-1.95	0.055	-3.94; -0.04	-0.15
	P	-2.51	0.013	-4.50; -0.53	-0.20

Total expectations → depression	A	-3.02	0.004	-5.07; -0.96	-0.24
	P	-1.95	0.055	-3.94; 0.04	-0.15

Indirect expectations → depression	A-A	-1.00	0.817	-12.44; 5.36	-0.08
	P-P	-0.07	0.719	-5.28; 1.61	-0.005
	A-P	0.30	0.943	-10.70; 6.80	0.02
	P-A	0.22	0.869	-4.67; 2.09	0.02

Total indirect expectations → depression	A	-1.07	0.003	-2.03; -0.32	-0.08
	P	0.52	0.210	-0.26; 1.46	-0.16

Estim.: unstandardized estimate; Stand.: standardized estimate.

**Table 3:** Effects of the mediation model testing the direct and indirect effects of anger-out in meeting expectations–depression relationship for partner (P) and actor (A).

Effect	Type	Estim.	*p*	95% CI	Stand.
Direct expectation → anger-out	A	-1.07	0.333	-3.24; 1.10	-0.08
	P	-3.01	0.006	-5.18; -0,84	-0.24

Direct anger-out → depression	A	0.14	0.046	0.003; 0.28	0.14
	P	-0.11	0.134	-0.24; 0.03	-0.10

Direct expectations → depression	A	-3.18	0.002	-5.28; -1.13	-0.25
	P	-1.70	0.103	-3.75; 0.34	-0.13

Total expectations → depression	A	-3.01	0.004	-5.06; -0.95	-0.23
	P	-2.02	0.054	-4.07; 0.03	-0.16

Indirect expectations → depression	A-A	-0.15	0.663	-6.43; 1.36	-0.01
	P-P	0.32	0.929	-9.33; 4.75	0.02
	A-P	0.11	0.790	-5.79; 1.66	0.009
	P-A	-0.43	0.889	-10.64; 4.04	-0.03

Total indirect expectations → depression	A	0.16	0.647	-0.51; 0.92	0.01
	P	-0.31	0.373	-1.07; 0.36	-0.02

Estim.: unstandardized estimate; Stand.: standardized estimate.

**Table 4:** Zero-order of the Pearson correlation including means and standard deviations for study variables.

	Gender	ESA	Anger-in	Anger-out	Depression
Gender	—	-0.157^∗^	-0.032	-0.173^∗^	-0.026
ESA		—	0.266^∗^	0.135^∗^	0.209^∗^
Anger-in			—	0.029	0.390^∗^
Anger-out				—	0.191^∗^
Depression					—
*M*	—	46.67	19.86	16.27	12.67
SD		8.69	4.76	4.15	4.02

^∗^ *p* < 0.05; gender coded: 1, woman; 2, man.

**Table 5:** Regression coefficients for moderation models predicting anger-in and anger-out. ESA was centred around the mean.

	Anger-in				Anger-out			
	Coeff.	SE	*p*	95% CI	Coeff.	SE	*p*	95% CI
Gender	0.06	0.08	0.458	-0.10; 0.22	-0.13	0.07	0.063	-0.27; 0.01
ESA	0.22	0.55	0.690	-0.86; 1.30	-1.28	0.48	0.008	-2.23; -0.33
Gender x ESA	0.07	0.06	0.257	-0.05; 0.19	0.15	0.05	0.006	0.04; 0.25
*R* ^2^ change	0.003				0.018			
*F*(1, 403)	1.29				7.60			

Coeff.: coefficient.

**Table 6:** Regression coefficient in the model predicting depression.

	Coefficient	SE	*t*	*p*	95% CI	*r*_partial_
ESA	0.04	0.02	1.93	0.055	-0.001; 0.08	0.09
Anger-in	0.16	0.04	3.72	<0.001	0.08; 0.25	0.17
Anger-out	0.30	0.04	7.78	<0.001	0.23; 0.38	0.36

## Data Availability

The data that support the findings are openly available at https://osf.io/xdmct/ (doi:10.17605/OSF.IO/XDMCT).

## Additional Points

*Code Availability*. Data obtained from this study were examined using IBM SPSS Statistics 27.
